# Decoding semantics across fMRI sessions with different stimulus modalities: a practical MVPA study

**DOI:** 10.3389/fninf.2012.00024

**Published:** 2012-08-24

**Authors:** Hiroyuki Akama, Brian Murphy, Li Na, Yumiko Shimizu, Massimo Poesio

**Affiliations:** ^1^Akama Laboratory, Graduate School of Decision Science and Technology, Tokyo Institute of TechnologyTokyo, Japan; ^2^Machine Learning Department, Carnegie Mellon UniversityPittsburgh, PA, USA; ^3^Centre for Mind/Brain Sciences, University of TrentoRovereto, Italy; ^4^Department of E&I S, Tokyo City UniversityYokohama, Japan; ^5^Department of Computer Science and Electronic Engineering, University of EssexColchester, UK

**Keywords:** fMRI, MVPA, GLM, machine learning, computational neurolinguistics, individual variability, embodiment

## Abstract

Both embodied and symbolic accounts of conceptual organization would predict partial sharing and partial differentiation between the neural activations seen for concepts activated via different stimulus modalities. But cross-participant and cross-session variability in BOLD activity patterns makes analyses of such patterns with MVPA methods challenging. Here, we examine the effect of cross-modal and individual variation on the machine learning analysis of fMRI data recorded during a word property generation task. We present the same set of living and non-living concepts (land-mammals, or work tools) to a cohort of Japanese participants in two sessions: the first using auditory presentation of spoken words; the second using visual presentation of words written in Japanese characters. Classification accuracies confirmed that these semantic categories could be detected in single trials, with within-session predictive accuracies of 80–90%. However cross-session prediction (learning from auditory-task data to classify data from the written-word-task, or vice versa) suffered from a performance penalty, achieving 65–75% (still individually significant at *p* « 0.05). We carried out several follow-on analyses to investigate the reason for this shortfall, concluding that distributional differences in neither time nor space alone could account for it. Rather, combined spatio-temporal patterns of activity need to be identified for successful cross-session learning, and this suggests that feature selection strategies could be modified to take advantage of this.

## Introduction

Over recent years, embodied theories of conceptual representation and language use (Barsalou et al., 1999) have challenged more classical symbolic accounts, particularly in their account of grounding—that is the mechanism in the mind of a language learner through which the abstract and usually arbitrary representations of language come to be associated with meanings out the world. BOLD activations which are independently known to be associated with a particular stimulus modality have been observed in response to different modalities—e.g., visual presentation of a manipulable object can elicit activity in motor regions (Pulvermüller, 2005), and auditory presentation of a concrete concept can activate areas in the visual pathway (Chao et al., 1999). However these broad patterns of neural activation cited in support of embodied theories are also consistent with more general mechanisms of spreading activation, and some may even be artifacts of experimental procedures (Mahon and Caramazza, 2008). It may also be appropriate to question some of our “ground-truth” assumptions about gross functional localization, when we consider that the same semantic category-specific activations can be seen in the “visual cortex” of congenitally blind participants, who lack any visual experience (Mahon et al., 2009).

In fact, the embodied and symbolic accounts may not necessarily be exclusive, as areas that show selectivity to perceptual processing in a particular modality may also perform other functions that have not yet been revealed by region-based analyses. For reasons of computational efficiency, it could make sense for a symbolic architecture to be arranged such that abstract properties are encoded in vicinity to the embodied activations to which they correspond. Similarly, it might be physiologically cheaper to use compact abstract representations for the default representation of concepts, while selectively recruiting embodied and perceptual detail as the activity or communicative task at hand demands.

Finer grained analyses of population encodings may be able to shed some more light on these questions than conventional contrastive analyses, which assume homogenous stimulus conditions and monotonic brain activations of a fixed scale and local topography (imposed both by spatial smoothing, and cluster-based evaluation of statistical power). Machine learning methods—often termed Multi-Voxel (or Multi-Variate) Pattern Analysis (MVPA), when applied to fMRI data—can discern more complex regularities in the data, such as small relative changes in activation across populations of voxels, in response to a range of conditions of interest. They are now becoming widely used in cognitive neuroscience, particularly for classifying higher cognitive states (Haxby et al., 2001; Spiridon and Kanwisher, 2002; Cox and Savoy, 2003; Mitchell et al., 2004; Davatzikos et al., 2005; Kamitani and Tong, 2005; LaConte et al., 2005; Haynes and Rees, 2006; Norman et al., 2006; O'Toole et al., 2007; Mourão-Miranda et al., 2008; Lee et al., 2009; Mur et al., 2009; Pereira et al., 2009, 2010; Raizada et al., 2010; Weil and Reesa, 2010), and using a variety of classification strategies (e.g., Support Vector Machines of various types; Bayesian methods; constrained linear and logistic regressions; *k* Nearest Neighbor). They have been used to classify trials of neural activity according to word, phoneme, and other linguistic categories (Mahon and Caramazza, 2010; Willms et al., 2011), and have been applied in particular to lexical semantics (Mitchell et al., 2008; Murphy et al., 2009, 2011, 2012; Chan et al., 2011; Pereira et al., 2011). Beyond demonstrating that brain activity can be linearly decomposed into a set of semantically interpretable basis images, Mitchell et al. (2008) and other work by the same lab (Wang et al., 2004; Shinkareva et al., 2008) established that this model can generalize across word sets, sessions, participants, stimulus modalities and languages.

Certainly such cross-learning is more challenging (Wang et al., 2004; Aron et al., 2006; Lee et al., 2009) and typically yields lower classification accuracies, perhaps due to differences in experimental paradigm, but also more prosaic discrepancies in the shape and timing of the BOLD responses across participants (Aguirre et al., 1998; Duann et al., 2002; Handwerker et al., 2004) and sessions (McGonigle et al., 2000; Smith et al., 2005). But assuming a shared semantic basis the similarity structure should show some consistency (Wang et al., 2004; Kriegeskorte and Bandettini, 2007a,b; Clithero et al., 2011; Haxby et al., 2011).

Returning to the question at hand here, if single concepts are activated via different modalities, a more sensitive analysis might reveal the finer grained population encodings that reflect activity that is specific to a particular presentation modality, and modality-neutral activity, including those specific to particular semantic categories. Considering embodied theories of semantic representations, based on sensory-motor systems, there may also be a further interaction with a particular orthography (Weekes et al., 2005). The written stimuli used here combine both Japanese scripts, *kanji* (ideograms whose forms have semantic content to a varying degree), and *kana* (which like other alphabets use arbitrary form-sound mappings). Note that it is widely accepted that the orthographic confounds (which are natural in Japanese with multiple writing systems—even flexibly and arbitrarily combining *kanji* and *kana* in a single word) share both semantic and phonological aspects without any problem.

In this paper we take a preliminary step in this direction, by examining the degree to which category-specific activations are shared across different stimulus presentation modalities. We present the same set of living and non-living concepts (land-mammals, or work tools) to the same cohort of Japanese participants, who perform a property rehearsal task (Mitchell et al., 2008) in two sessions: the first using auditory presentation of spoken words; the second a matter of days or weeks later, using visual presentation of words written in Japanese characters.

We first use a cross-validated classification strategy to identify the semantic category (mammal or tool) of single stimulus trials. A univariate feature-selection is used in conjunction with a regularized logistic regression classifier to reliably isolate the subset of voxels that are more informative for distinguishing between these two stimulus types. This single-participant, uni-modal analysis, together with a conventional General Linear Model (GLM) analysis, establish that the data correspond to established patterns familiar in the literature, and that our data contains enough information to discriminate these semantic classes. Next we attempt to decode category across modalities: that is by training on auditory stimulus data, and classifying orthographic stimulus data; or vice versa. While both yield highly significant classification accuracies, there is a clear performance penalty for cross-modal classification relative to uni-modal analysis. We perform several follow-on analyses to investigate whether this penalty is due to differences in timing, in location, or due to varying temporal-coding within similar regions.

## Materials and methods

In many respects our experimental paradigm replicates Mitchell et al. (2008), especially in that we adopt the same behavioral task which asked participants to silently rehearse semantic properties on presentation of the stimulus, the same slow event-related design, and the same principal scanning settings with a coarse whole brain image (3 × 3 × 6 mm) at a short TR of 1 s.

Participants completed two sessions, first viewing pictures while listening to the spoken word describing the represented object (the auditory condition), and next viewing pictures with an accompanying caption (the orthographic condition). They were asked to silently enumerate properties that are characteristic of the presented concept. The instructions actually used in the experiments are given in the supplementary materials.

Our analysis of the data used a category-decoding task, predicting if each trial presented an animal or artifact stimulus. The initial unimodal analysis was followed by a cross-modal analysis to identify the extent to which activations are shared across the two modalities of presentation. Follow-on analyses that varied the selection of temporal and spatial input were used to further elucidate the more limited overlaps in activations observed.

### Materials

Each of the participants was presented on screen with a series of contrast-normalized gray-scale photographs of tools and mammals, using the E-Prime 2.0 software package. These items were selected from a set of stimuli previously used for predicting EEG activation patterns (Murphy et al., 2009, 2011). Twenty stimuli in each of the two classes were presented in random order without repetition in each run, and this presentation of 40 stimulus items was repeated six times, for a total of 240 trials. The same images were accompanied by the spoken presentation of their Japanese name in the first auditory condition session, and by a Japanese caption in the second orthographic condition session. Each trial was presented visually for 3 s, and in the auditory condition the spoken name started simultaneously with a visual onset and lasted approximately 2 s on average. Participants were asked to recall a word which represented a typical attribute or property of the object during the 3 s of the visual stimulus, which was followed by a 7 s rest period, during which the subjects were asked to fixate on cross mark displayed in the center of the screen. There were six additional presentations of a fixation cross, 40 s each, distributed just after each run to establish a BOLD baseline.

The concepts used were:

**Mammals:** *anteater (*
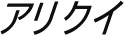
*), armadillo (*
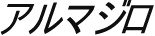
*), beaver (*
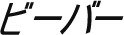
*), camel (*
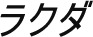
*), deer (*
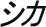
*), elephant (*
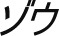
*), fox (*
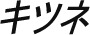
*), giraffe (*
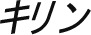
*), gorilla (*
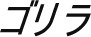
*), hare (*
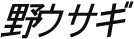
*), hedgehog (*
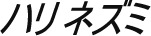
*), hippopotamus (*
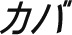
*), kangaroo (*
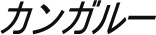
*), koala (*
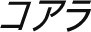
*), mole (*
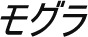
*), monkey (*
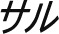
*), panda (*
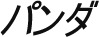
*), rhinoceros (*
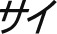
*), skunk (*
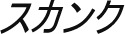
*), and zebra (*
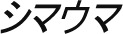
*)*.

**Tools:** *Allen key (*
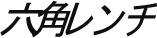
*), axe (*
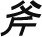
*), chainsaw (*
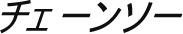
*), craft knife (*
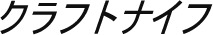
*), file (*
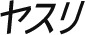
*), hammer (*
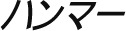
*), nail (*
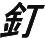
*), paint roller (*
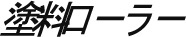
*), plaster trowel (*
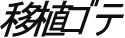
*), pliers (*
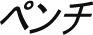
*), plunger (*
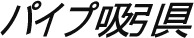
*), power drill (*
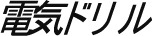
*), rake (*
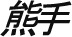
*), saw (*
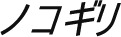
*), scraper (*
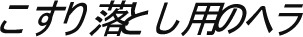
*), scissors (*
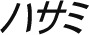
*), screw (*
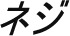
*), sickle (*
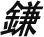
*), spanner (*
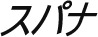
*), and tape measure (*
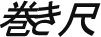
*)*.

### Participants

Six volunteers (4 males, 2 female, age range 39–53 years) were recruited and scanned. All subjects were native-Japanese speaking subjects, right-handed and had no known history of neurological impairment. Ethical approval was obtained from the local Human Investigation Committee of the Graduate School of Decision Science and Technology at Tokyo Institute of Technology and the volunteers signed a written informed consent form. They were asked to perform an off-line property generation task for all items before each fMRI session, and reported refraining from coffee and alcohol from one night before. One subject was dropped from the study due to excessive movement (>2 mm) during the first auditory session. The remaining five (P1, P2, P3, P4, and P5) subsequently completed the second scanning session using the other stimulus modality condition (the orthographic condition).

### Imaging techniques

Images were obtained using a 3.0-T General Electric Signa scanner at Tokyo Institute of Technology, Japan with a 8-channel high resolution head coil. Scanning parameters were based on those of Mitchell et al. (2008). Functional scanning was performed using an echo planar imaging sequence with a 1000 ms repetition time (TR), 30 ms echo time (TE), and 60° flip angle (FA). Each volume consisted of 15 × 6 mm thick slices with an interslice gap of 1 mm; FOV: 20 × 20 cm; size of acquisition matrix, 64 × 64; NEX: 1.00. The parameter values of the anatomical scans were TR = 7.284 ms, TE = 2.892 ms, FA = 11 degrees, Band Width = 31.25 kHz, voxel size = 1 mm isotropic. Following settings used by Mitchell et al. (personal communication), we set oblique slices in the sagittal view with a tilt of −20° to −30° such that the most inferior slice is above the eyes anteriorly and passes through the cerebellum posteriorly.

### Preprocessing and general linear model (GLM)

The fMRI data were preprocessed and analyzed using Statistical Parametric Mapping software, SPM8 (version 4290) (Friston et al., 1995). Pre-processing steps included motion correction, coregistration of functional and anatomical images, segmentation to identify grey matter, and normalization into standard Montreal Neurological Institute (MNI) space at a resliced voxel size of 3 × 3 × 6 mm. Further details of the SPM settings used are given in the supplementary materials.

For the GLM analysis the data was additionally smoothed using an 8 mm Gaussian kernel. A conventional GLM contrastive analysis was first performed as a data-validation step. Single-session analyses were made on four contrasts with FWE-adjusted *p* < 0.05: task > rest; rest > task; mammal > tool; and tool > mammal. A random effects analysis was also executed with respect to the 10 datasets of the five participants to confirm the tendencies found in each single subject analysis.

### Multi variate pattern analysis (MVPA)

Machine-learning analyses were performed using the PyMVPA0.6 package (http://www.pymvpa.org/; Hanke et al., 2009). The realigned, coregistered and normalized (but unsmoothed) functional images of each subject in each session were loaded and filtered according to the grey matter mask (i.e., non-cortical voxels were ignored) and were used for cross-validated trial-wise training and testing of the animal/tool distinction. As each stimulus was presented six times, six-fold cross-validation was used for the unimodal analysis: each stimulus was represented by exactly five trials in the training partitions, and by one test trial in the evaluation partitions, and each trial was tested exactly once. The classification accuracies reported are the simple mean of the 240 trial classification results (1 for correct; 0 for incorrect), which represents the proportion of trials whose semantic category was recognized correctly. For the cross-modal analysis the data was partitioned such that training was performed exclusively on the data of one modality, and testing on the other modality. Feature selection was performed strictly within the training partitions, taking the top 500 voxels according to an ANOVA-based ranking.^1^

In terms of the choice of classification algorithm, functional MRI data is typically noisy, highly redundant, and has a large number of features relative to the number of training examples. In this study we aim to balance successful classification with sufficient interpretability, in terms of being able to identify which voxels/regions are informative for the distinction of interest. The penalized logistic regression (PLR) classifier we selected is well-suited since its regularization term deals with both high dimensionality and redundancy in data by spreading the learning load over groups of similar voxels; its logistic function is optimized to fit discrete data categories; and it makes similar assumptions of linearity to those of a GLM.^2^

More precisely, the classifier uses *L2*-norm regularization (also termed ridge regression or Tikhonov regularization) by defining a penalty term to minimize the sum of the squared beta values (1), with a tuning parameter λ (here set to 1.0). This has the effect of sharing the distribution of learning weights *w* over *X*, the set of BOLD magnitudes recorded at each selected voxel. The discrete nature of the dependent variable (in our case, the category of animal vs. tool) is modeled with the logarithm of the odds ratio (2).

(1) $$y\;=\;wX\;+\;\lambda w^{2}$$

(2) y =log[p/(1−p)]

Additional preprocessing steps that applied to the multi-variate analysis consisted of linear detrending and *z*-score normalization of each voxel time course to control for both global and local variations in baseline haemodynamic response. Trial-level images were computed by taking a simple average of four consecutive fMRI volumes, offset by 4 s from the stimulus offset (termed a “boxcar” model of the BOLD response; cf. Mitchell et al., 2008). Preliminary analyses indicated that this common averaging strategy was similarly successful to using a HRF-model-weighted average.

## Results

### GLM

**Figure 1:**
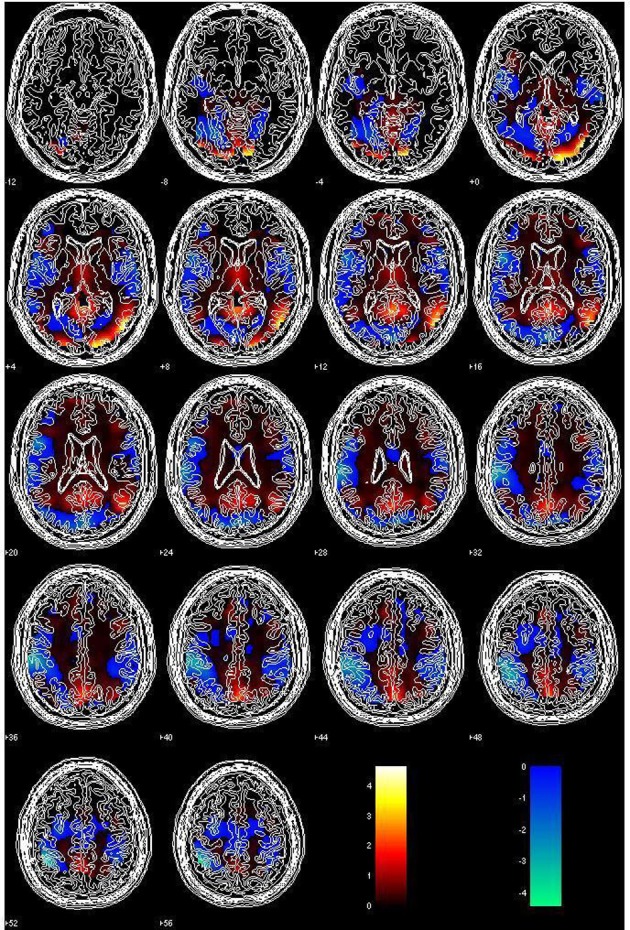
**The activation maps of the two contrasts (hot color: mammal > tool; cool color: tool > mammal) computed from the 10 datasets of our participants**. The apparently sharp cutoff of values in the most ventral slices was not due to the mismatch with the contours of the normalized space, but to the relative narrowness and the shape of the coverage extent (due to only 15 oblique slices as the result of TR = 1 s), which was the logical AND of the individual coverage spheres.

The activations identified in the GLM analysis at the first and the second levels were approximately consistent with established areas of animal and tool specificity (Chao et al., 1999; Pulvermüller, 2001; Binder et al., 2009). Figure 1 represents a series of transversal slices combining the activation maps of the two contrasts (mammal > tool and tool > mammal) used for the random effects analysis of variance with GLM (*p* < 0.005, unadjusted) applied to the data of the five participants. As classification accuracy is a primary goal of our MVPA study, we chose a high temporal resolution (i.e., a shorter TR = 1 s, allowing us to include many time points), at the expense of spatial resolution, achieved with thick slices to still cover the great majority of cortex.

According to the output T-contrast of mammal > tool in our study, the mammal items, whose visual complexity was significantly higher than the other semantic category, showed a large area of strong activation in right temporal and occipital lobes (visual area). On the other hand, the tool area could be identified in left inferior parietal lobe and supra-marginal gyrus; more precisely, several loci in the sensory-motor area, which were related to the tactile images on right fingers, were marked for some participants in the parametrical maps of the T-contrast of tool > mammal, showing a tendency which was in line with the simulation semantics or embodiment theory (Barsalou, 1999, 2003; Glenberg and Kaschak, 2002; Vingerhoets et al., 2002; Feldman and Narayanan, 2004; Bergen, 2005; Rohrer, 2007; Wu and Barsalou, 2009; Devereux et al., 2010; Willems and Casasanto, 2010).

### Uni-modal classification

**Figure 2:**
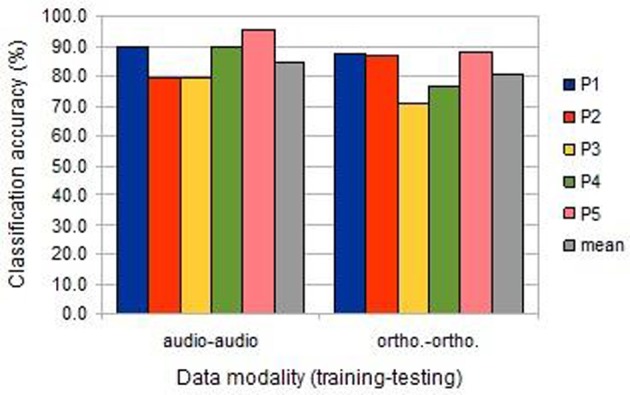
**The classification accuracies obtained under the within-session uni-modal conditions from the five participants (BOLD delay = 4; number of volumes = 4)**.

Animal vs. Tool classification accuracy was computed individually using the data from each participant session. As there were 240 cases in this experiment, classification accuracies above 55.8% are significantly higher than expected by chance (at *p* < 0.05, binomial test, chance 50%, *n* = 240). As is clear from Figure 2, classification was highly significant for all session analyses, both in the image/auditory condition (“audio-audio”) and the image/orthographic condition (“ortho-ortho”). For all participants, save the second, classification was more successful in the auditory condition.

### Cross-modal classification

**Figure 3:**
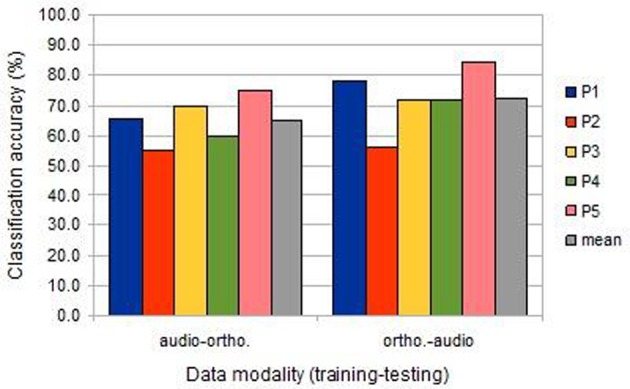
**The classification accuracies obtained under the inter-session cross-modal conditions from the five participants (BOLD delay = 4; number of volumes = 4)**.

Compared to uni-modal classification, the inter-session cross-modal prediction was somewhat less successful, but with a similar ranking among participants (Figure 3). All results were significantly above chance, except for participant 2 when training on the image/auditory data and testing the image/orthographic data. This “audio-ortho” direction of learning proved less successful generally than the “ortho-audio” direction.

### Discussion and follow-on analyses

#### Accounting for the cross-modal penalty

While the predictive analysis provided highly significant results, there appeared to be systematic variation in the different learning conditions: within sessions, auditory data was easier to classify than orthographic data; within session classification was easier than cross-session classification; and training on orthographic condition data and testing auditory data was easier than vice versa. We conduct several follow-on fitting analyses investigate the reasons for these trends, concentrating on temporal and spatial variations in haemodynamic response.

#### Development of classification accuracy over time

**Figure 4:**
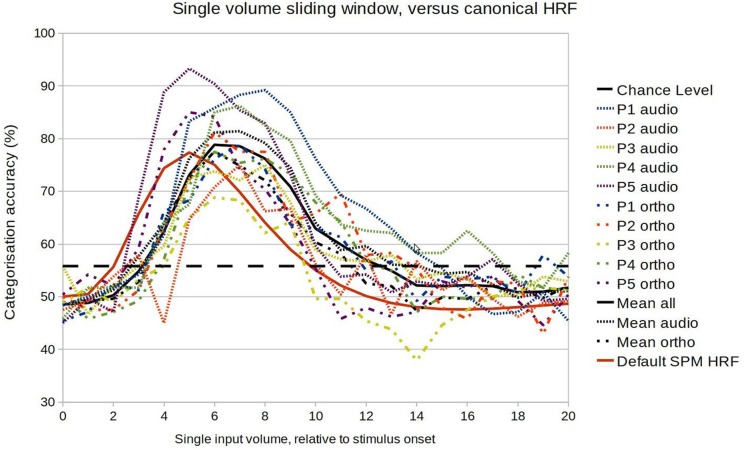
**Comparison between the model accuracy function and the canonical HRF in the range of 0–20 s after stimulus onset**.

We first performed a fine-grained examination of the temporal development of classification accuracy. Within-session classification was performed exactly as before except that the input was a volume at a time, at increasing latencies relative to the stimulus onset. This should reveal similar general patterns to the previous analysis, but with lower accuracies overall (as the algorithm has less data to learn from). As Figure 4 indicates, there was considerable variation in the temporal development of classification accuracy (i.e., in the amount of information encoded in the selected voxel population), with all showing a profile typical of a HRF response. The mean profile corresponded approximately to a gamma function with parameters peak at 7 s, and FWHM (Full width at half maximum) of 6 s, a response somewhat later than is usually assumed. However there did not seem to be any systematic difference in patterns between the auditory and orthographic conditions. The main regularities were specific to participants, with correlations between the profiles of both conditions of 0.88 for P1, 0.72 for P2, 0.81 for P3, 0.91 for P4, and 0.96 for P5.

**Figure 5:**
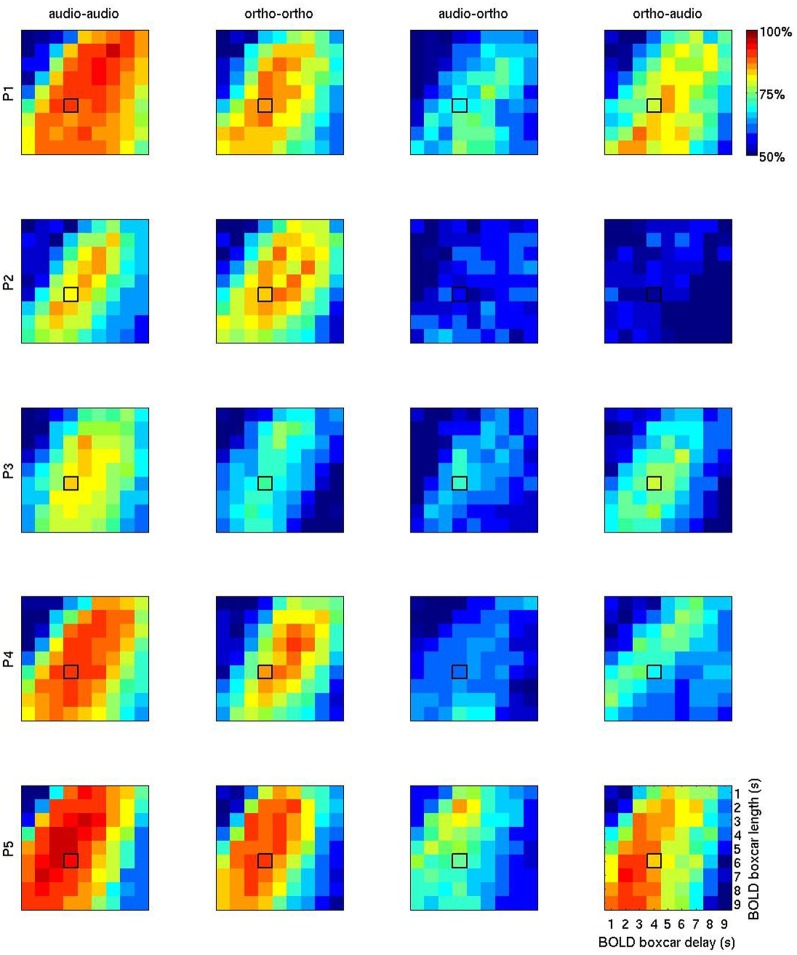
**BOLD accuracy grids containing the overall results of 1620 (= 9 × 9 × 4 × 5) machine learning computations using PLR**. The first two columns (“audio-audio” and “ortho-ortho”) stand for the results of the within-session uni-modal predictions for P1 (row 1), P2 (row2), P3 (row3), P4 (row4), and P5 (row5). The columns 3 (“audio-ortho”) and 4 (“ortho-audio”) are for the results of the inter-session cross-modal prediction. “audio” and “ortho” stand for auditory and orthographic conditions, respectively. The horizontal axis represents the BOLD delay relative to stimulus onset (1–9 s) and the vertical one number of volumes, or width (1–9 s). The initial default boxcar parameters (delay = 4 s, width = 4 s) is outlined in black on each plot.

Figure 5 shows a more exhaustive grid search of the optimal BOLD boxcar averaging parameters, taking an onset delay of 1–9 s and a boxcar width from 1 to 9 s. Looking at the first two columns of unimodal results, the temporal patterns are broadly similar to those seen in the Figure 4. The initial choice of boxcar (delay = 4 s; width = 4 s) appears close to the optimal in many cases, though for participants 1, 2, and 3 later and longer windows might have proved slightly more effective. Again, the largest variations seem to be between participants, rather than across modalities, so this alone cannot explain the observed cross-session penalty.

The patterns in the two rightmost columns are very different. Apart from the general reduction in classification accuracy, the optimal regions for cross-modality modeling overlap with those seen for unimodal modeling. But between the two cross-modal conditions the most informative temporal regions are often disjoint. For example in participant 4, the most informative volumes for using auditory condition data to learn about orthographic condition data (“audio-ortho”) are earlier and shorter than those for the “ortho-audio” direction. This is counter-intuitive, since patterns that generalize well in one direction might be expected to work similarly well in the opposite direction.

#### Spatial distribution of informative voxels

**Figure 6:**
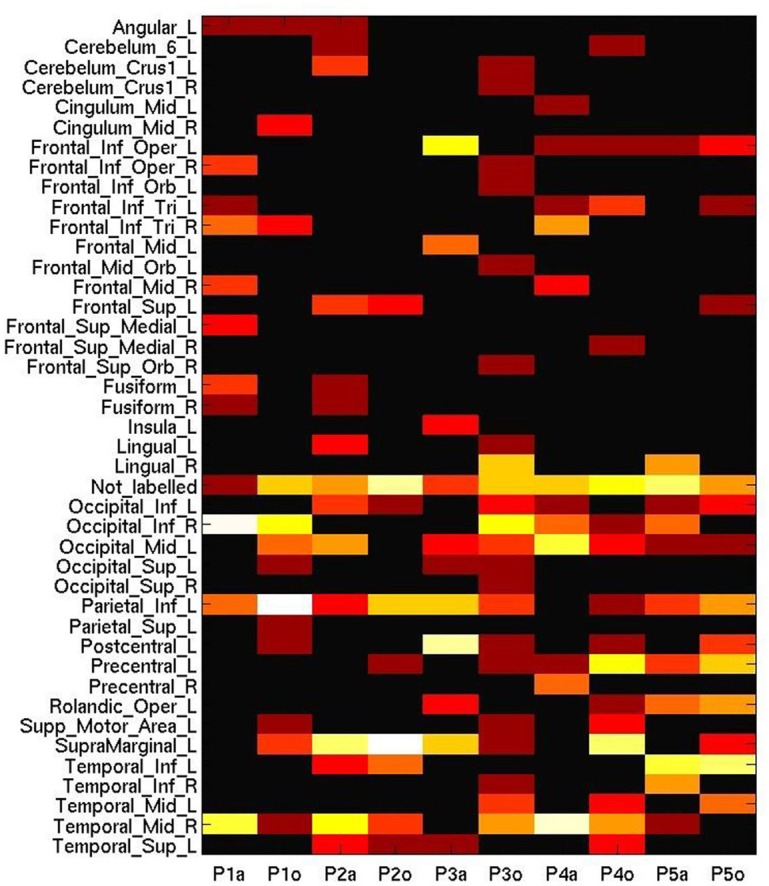
**Number of most informative voxels extracted by anatomical area (AAL brain atlas), ranging from 0 (black) to 20 (white), on a log-adjusted scale**. Columns represent participant numbers, and stimulus modality (“a”, auditory; “o”, orthographic).

An alternative explanation for these discrepancies would be differences in the anatomical distribution of the informative voxels which encode semantic category in each of these two modality conditions. In this respect MVPA is typically more sensitive to local topographical coding of information than a GLM, which is conversely more sensitive to global engagement (Jimura and Poldrack, 2011). Figure 6 displays the AAL (Anatomical Automatic Labeling) regions of interests to which are roughly attributed the 50 voxels that the process of machine learning evaluated as the most sensitive and informative features for the cross-session multi-modal prediction in the predictive analysis. Note that the localization was at a coarse grain as a result of the scanning parameters, that traded off a voxel size of 3 × 3 × 6 for a higher temporal resolution (TR = 1 s). Here we do see variation across participants in the most informative loci, such as right temporal and occipital lobes (visual area), left inferior parietal lobe and supra-marginal gyrus, which approximately accord with the GLM results.

However, it could be recognized in MVPA that the auditory session triggered a biased sensitivity to many voxels in the frontal, occipital and temporal lobes, whereas the areas generally considered as discriminative for processing tool words, such as the left inferior parietal lobule or the left supramarginal gyrus, were more engaged in the orthographic condition (with the exception of P3). Moreover, despite the scattering of these informative voxels across wide-ranging brain areas, Figure 6 does not include the labels corresponding to some peaks (*p* < 0.001, unadjusted) elicited by the second level analysis of GLM (Occipital_Mid_R, Precuneus_L). This comparison might suggest the insufficiency for cross-session analysis of placing undue reliance on any spatial pattern based on well-established functional anatomy.

### Spatio-temporal correlation across modalities

The final follow-on analysis investigates whether a combination of spatial and temporal alignment (or lack there-of) between the haemodynamic responses gives an account for the variation in cross-modal classification performance. Event-related average responses were calculated for each session over 11 s of data from each trial epoch. This was done over the selected informative voxels for the session in question, and separately for each semantic category. The final measure of fit between two sessions was quantified as the cosine similarity between the vectors of the mammal/tool difference time-courses. Since the voxels considered depended on the feature selection step of the training data, different measures were produced depending on the direction of the training and testing.

**Figure 7:**
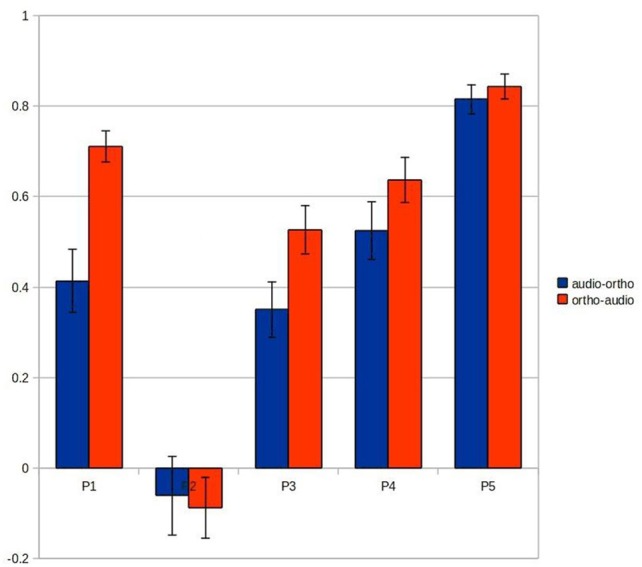
**Correlations between the vectors of the mammal/tool difference time-courses recorded at the voxels selected for the audio-ortho (blue) and ortho-audio predictions (red)**. Each error bar represents Standard Error.

Figure 7 shows the results of this analysis and suggests that this spatio-temporal fit may account for much of the variation in main results (Figure 3). The “ortho-audio” task, which had uniformly higher classification accuracies, also shows higher correlations on this analysis, and the one participant with clearly worse performance (the second) has negligible correlations, but with a standard deviation above chance.

## Conclusion

In this study, we showed that an appropriate machine learning technique was able to learn category specific codings successfully, which generalized across stimulus modalities. Within-session uni-modal prediction achieved accuracies in 80–90% range for discriminating the semantic categories of stimuli. Cross-modal classification (range 65–75%) was also highly significant, but suffered from a clear performance penalty relative to unimodal analyses.

However follow-on fitting analyses revealed substantial differences in development of the haemodynamic response, relative to commonly assumed standard models, and more concretely between the participants included in this study, and further across sessions from a single participant. This leads to several observations that may have methodological consequences: (1) boxcar averaging of BOLD data can be an effective and faithful approximation of more common models such as a gamma function; (2) model accuracy function in MVPA was *isomorphic* to a particular basis function for the BOLD effect; (3) successful cross-session learning relies on spatio-temporal correlations in class-specific activity. This suggests that an effective feature selection strategy would be to identify voxels across sessions that are both responsive to the different classes of interest (supervized, using the labeled training data within cross-validation) and which show considerable correlation in their temporal profile between the training and test datasets (unsupervized, irrespective of class labels). With the data we present in the current study, it would not be possible to test this hypothesis without running the risk of overfitting by “double-dipping” (Kriegeskorte et al., 2009). However these results suggest that voxel-specific adaptive selection of BOLD modeling parameters, and feature selection strategies based on spatio-temporal correlations, may provide substantial advantages in classification accuracy, also in real-world applications of these technologies, such as brain-computer-interfaces for communicatively impaired patients.

### Conflict of interest statement

The authors declare that the research was conducted in the absence of any commercial or financial relationships that could be construed as a potential conflict of interest.

## Footnotes

^1^Under certain conditions, linear machine learning algorithms (such as PLR) can be relatively insensitive to the number of features selected—cf. Table 1 of Pereira et al., 2009. Previous parameter exploration analyses we have carried with similar datasets (same stimuli, similar task, different languages, identical analysis) indicated that the number of selected features could range from 50–5000 without substantial differences in performance.

^2^*L2* regularization is appropriate in that it spreads the learning load over groups of co-varying voxels. While an *L1*-regularised model might conceivably provide higher classification accuracies, this would be at the cost of partly arbitrary feature selection (in the sense that one voxel from a group of co-varying voxels might be chosen for reasons as uninteresting as the fact that it has a lower level of extraneous noise). Similarly, the margin maximization criterion of an SVM could also improve learnability, but it is not obvious to us how this criterion is coherent with common assumptions about neural coding and functional localization.
